# From Prebiotic Chemistry to Supramolecular Biomedical Materials: Exploring the Properties of Self-Assembling Nucleobase-Containing Peptides

**DOI:** 10.3390/molecules26123558

**Published:** 2021-06-10

**Authors:** Pasqualina Liana Scognamiglio, Chiara Platella, Ettore Napolitano, Domenica Musumeci, Giovanni Nicola Roviello

**Affiliations:** 1 Center for Advanced Biomaterial for Health Care (CABHC), Istituto Italiano di Tecnologia, I-80125 Naples, Italy; pasqualina.scognamiglio@iit.it; 2 Department of Chemical Sciences, University of Naples Federico II, via Cintia 21, I-80126 Naples, Italy; chiara.platella@unina.it (C.P.); ettore.napolitano@unina.it (E.N.); domenica.musumeci@unina.it (D.M.); 3 Istituto di Biostrutture e Bioimmagini IBB-CNR, via Tommaso De Amicis 95, I-80145 Naples, Italy

**Keywords:** peptides, nucleobases, bioactivity, nucleopeptides, PNAs, nucleic acids, prebiotic, self-assembly, nanomaterials, diagnostic, therapeutic, supramolecular

## Abstract

Peptides and their synthetic analogs are a class of molecules with enormous relevance as therapeutics for their ability to interact with biomacromolecules like nucleic acids and proteins, potentially interfering with biological pathways often involved in the onset and progression of pathologies of high social impact. Nucleobase-bearing peptides (nucleopeptides) and pseudopeptides (PNAs) offer further interesting possibilities related to their nucleobase-decorated nature for diagnostic and therapeutic applications, thanks to their reported ability to target complementary DNA and RNA strands. In addition, these chimeric compounds are endowed with intriguing self-assembling properties, which are at the heart of their investigation as self-replicating materials in prebiotic chemistry, as well as their application as constituents of innovative drug delivery systems and, more generally, as novel nanomaterials to be employed in biomedicine. Herein we describe the properties of nucleopeptides, PNAs and related supramolecular systems, and summarize some of the most relevant applications of these systems.

## 1. Introduction

**Figure 1:**
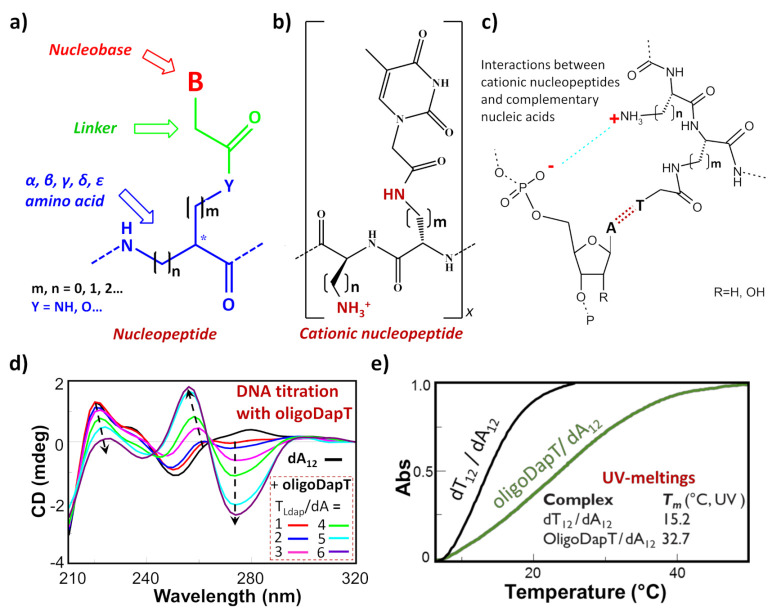
(**a**) Schematic representation of a generic nucleopeptide; (**b**) repetitive unit of a generic cationic nucleopeptide with backbone composed of free and nucleobase-bearing diamino acids; (**c**) H-bonding and ionic interactions between cationic nucleopeptides and complementary nucleic acids; (**d**) example of CD titration of a single-stranded DNA (dA_12_) with a complementary homothymine nucleopeptide based on a L-diaminopropanoic acid (DAPA) backbone (oligoDapT) [1]; (**e**) comparison of melting curves and temperatures between the natural duplex DNA and the corresponding oligoDapT-nucleopeptide/DNA complex [1]. Atom with the symbol “ * ” represents a stereogenic center.

Among the nucleic acid mimetics, nucleopeptides—i.e., natural or synthetic compounds composed of nucleobases inserted on a peptide backbone (Figure 1a)—show interesting features deriving from their experimentally proven capacity to bind complementary RNA and DNA sequences [1,2,3,4,5,6,7,8,9]. The H-bonding ability between the complementary bases of the nucleic acid and the nucleopeptide can be reinforced by other kinds of binding interactions, such as the electrostatic ones (Figure 1b,c). This is the case, for example, of cationic nucleopeptides obtained by the sequential oligomerization of diamino acids, in the form of nucleobase-bearing and base-free units (Figure 1b) [1,2,4]. Specifically, in various recent papers [1,3,4], the electrostatic contribution to the interaction of a cationic nucleopeptide with nucleic acids was qualitatively measured by circular dichroism (CD) and UV experiments, denoting that the binding with DNA and RNA was mainly directed by complementary base–base recognition (Figure 1d,e).

In addition, polyamino acid chains carrying even a single nucleobase-bearing amino acid (also referred to as a nucleoamino acid) were proven to interact, in some cases conferring stabilization, with various protein and peptide structures, with a consequent interference in their functions [10,11,12,13,14,15]. Indeed, enzymatically stable mononucleobase-containing dipeptides were found to bind serum albumins [15], whereas a single thymine-bearing nucleopeptide has been shown to interact with the reverse transcriptase (RT) of Human Immunodeficiency Virus (HIV), leading to inhibition of its activity [11]. The ability of nucleopeptides to specifically recognize proteins has been also explored in the case of the Moloney Murine Leukemia Virus (M-MLV) RT [10] and of SARS-CoV-2 M^pro^ through in silico studies [14].

Nucleopeptide monomers, i.e., nucleoamino acids [9], can be (i) naturally occurring compounds such as willardiine and others [16,17,18,19], (ii) mimics of natural molecules, or (iii) synthetic building blocks. Since modified nucleosides containing nucleobase modifications are interesting scaffolds featured by several biological properties, e.g., antiviral or antitumor activities [20,21,22,23,24,25,26,27], the wide range of these modified nucleobases can be combined with amino acidic units to obtain new bioactive nucleoamino acids, also constituting useful synthetic building blocks for novel nucleopeptides.

**Figure 2:**
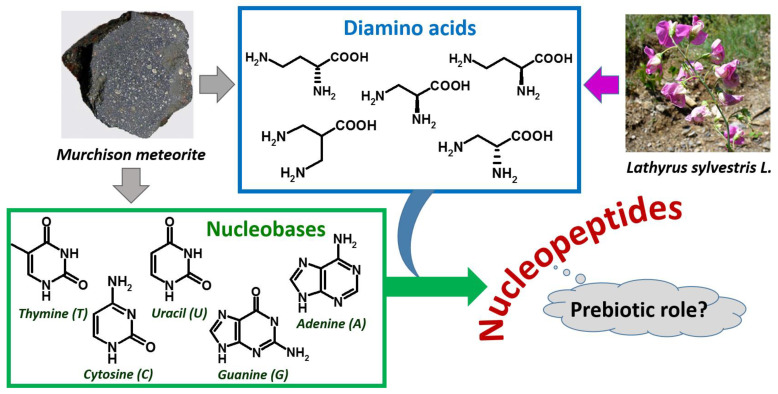
Extraterrestrial and plant sources of diamino acids and nucleobases as components of nucleopeptides, hypothesized to be involved in a prebiotic scenario.

As far as the amino acid composition is concerned, nucleopeptides can include both proteinogenic and non-proteinogenic α-amino acids [1,3,28,29,30,31,32,33,34]. Some examples of nucleopeptides with backbones based on non-proteinogenic diamino acids with interesting properties are those based on the plant-occurring L-diaminobutanoic acid (DABA, Figure 2) and L-diaminopropanoic acid (DAPA); interestingly, their diamino acid units were found in vegetal sources and various microorganisms (Figure 2) [35,36,37].

It is worth mentioning that, even though, to the best of our knowledge, no examples of nucleopeptide sequences fully based on nucleoamino acids have been found in natural sources to date, excluding the short willardiine nucleopeptides [38], no other DNA analog has monomers of natural origin.

In addition to targeting complementary DNA and RNA sequences, nucleopeptides are endowed with further biomedically relevant properties, as they are able to self-assemble forming supramolecular networks [39,40], they can cross cellular membranes [41,42] and exert other important functions, such as the delivery of small molecules and oligonucleotide-based drugs [43,44,45,46].

In this overview, some of the most inspiring aspects of nucleopeptide self-assembly have been summarized in the context of prebiotic and supramolecular chemistry, also focusing on their potential applications in biomedicine and nanotechnology.

## 2. Self-Assembling Nucleopeptides as Potential Prebiotic Genetic Materials

Prebiotic chemistry, one of the most exciting topics in the field of research on the chemical origins of life, suggests that some of the precursors of nucleic acids and proteins might be formed in prebiotic environments [47,48].

The isolation of DABA and other diamino acids, as well as certain nucleobases, in the meteoritic soil coming from the Murchison meteorite [49], together with the absence of nucleic acid-forming sugars in the same specimens [50,51], led to hypothesize that the prebiotic world was founded on nucleopeptides (Figure 2) [49,52,53,54].

**Figure 3:**
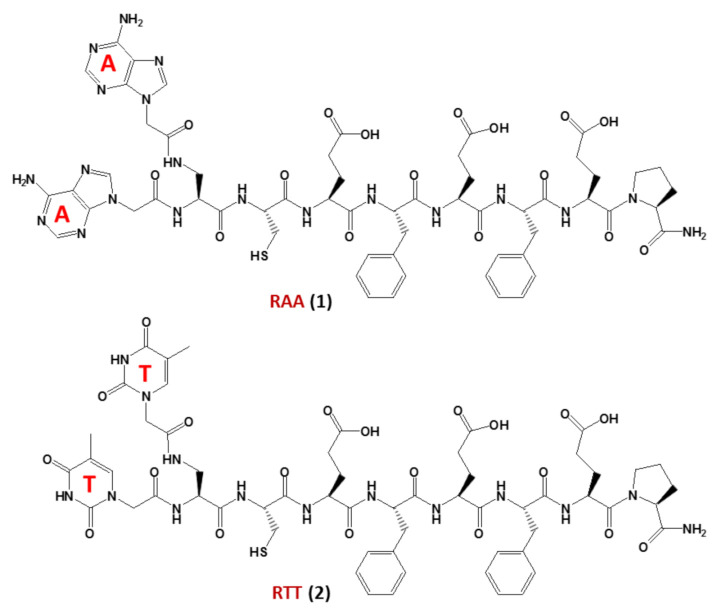
The nucleopeptide chimeras RAA (**1**) and RTT (**2**), investigated as non-enzymatically self-replicating prebiotic genetic materials by Bandela et al. [57].

In detail, nucleopeptides could be implied in a prebiotic scenario preceding the “RNA world” [55], in which they acted as self-replicating molecules. The interaction between nucleopeptides and RNA could have played a pivotal role [56] in the transition from the “nucleopeptide world” [53] to the current genetic system based on DNA, RNA and proteins. In this regard, studies on non-enzymatic replication—a central mechanism driving chemical evolution—focused recently on nucleopeptides using chimeric nucleobase-peptide derivatives (**1** and **2**, named RAA and RTT, respectively, Figure 3). It was proven that different mechanisms control the replication of complementary nucleopeptides with a clear selection of one structure over the others [57], leading to the hypothesis that similar processes may have been the origin of the first functional peptide-nucleic acid assemblies, which in turn led to the appearance of biological assemblies such as the ribosomes. In particular, the self-organization and selection processes were shown to occur in mixtures containing short complementary nucleopeptides composed of eight α-amino acids conjugated at their *N*-termini, through carboxymethylene linkers, to adenines or thymines in RAA (**1**) and RTT (**2**), respectively (Figure 3). As shown by both experimental and simulation studies, the autocatalytic and cross-catalytic template-directed replication processes, which were synergistically governed by both A:T hybridization and the formation of supramolecular architectures of the peptide segments of each nucleopeptide, occurred within these nucleopeptide networks determining the product formation [57].

In summary, nucleopeptides are believed to have acted as prebiotic genetic materials, not only due to the extraterrestrial occurrence of their chemical constituents, but also because, through their self-organization governed by both hybridization of nucleobase motifs and the self-assembling propensity of the peptide segments, they could self-replicate even in the absence of enzymes facilitating the process. These findings are suggestive because they are clear evidence in support of the putative crucial role of nucleopeptides in directing the transition from non-living matter to primitive life [57].

## 3. Self-Assembling Guanine-Bearing Nucleopeptides and PNAs: G-quadruplex (G4) Structure Formation

Among the unusual nucleic acid secondary structures, the G-quadruplex (G4) family is one of the most intriguing and widely investigated [58,59,60,61,62,63]. It has been demonstrated that G4 DNA and RNA play crucial roles in many physiological and disease-related biological mechanisms. G4-forming sequences are indeed present in several important regions of the human genome, including telomeres [64], gene promoters [65] and the 5′-untranslated region (5′-UTR) of RNA [66].

Significant efforts have been devoted to the study of G4s in telomeric regions because their formation can interfere with crucial physiological processes generating telomere instability. The formation of a G4 can represent an obstacle for DNA polymerase which, if not removed, could cause double-strand breaks in subsequent replications [67]. Moreover, the presence of G4 can also inhibit telomerase activity, inducing telomere shortening [68].

On the other hand, the selective stabilization of telomeric G4 in cancer cells can downregulate the telomerase, representing a promising anticancer strategy [69]. This enzyme is indeed overexpressed in several types of tumors and is responsible for the “immortality” of tumor cells [70]. In this context, targeting G4 structures or using G4 forming aptamers able to bind specific targets involved in pathological conditions are exciting strategies with the potential to control gene expression and design anticancer and antiviral therapeutics [65,71,72,73,74,75,76,77,78,79,80,81,82,83]. Apart from the large number of guanine-rich (G-rich) natural oligonucleotides forming G4 DNA and RNA structures, —PNAs (peptide nucleic acids)—that are oligomers with pseudopeptide backbones bearing nucleobases—were also shown to form intermolecular G4s endowed with the structural features of a natural G4, but without the anionic DNA (RNA) backbone, as proven by the combined use of mass spectrometry and spectroscopic methods [84]. Other G4 structures were observed, with the PNA sequence G_4_T_4_G_4_ involving both DNA–PNA hybrid structures (PNA_2_−DNA_2_ G4) [85] and pure PNA self-assemblies including both dimeric and tetrameric G4s, with a preference for the former [86]. PNAs were also found to form bimolecular [87] and unimolecular antiparallel G4 [88]. G-rich PNAs bind to homologous nucleic acids to form hetero-G4s, but can also bind to complementary cytosine-rich DNA and RNA sequences to form hetero-duplexes. The incorporation of chiral modifications (nucleoamino acids), as well as of abasic sites, into the backbone of G-rich PNAs was a valid strategy for improving selectivity for the hetero-G4 vs. hetero-duplex formation. This was explained by the significant decrease of affinity for complementary sequences accompanied by only slight effects on the high-affinity binding to the homologous nucleic acids to form hetero-G4s [89].

**Figure 4:**
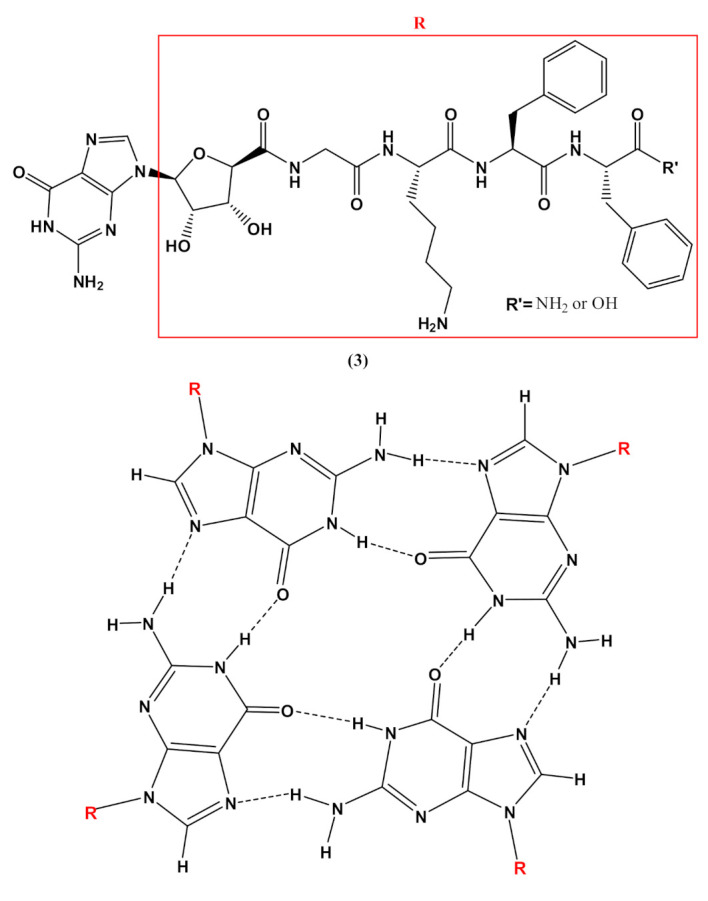
Chemical structures of the nucleo-tetrapeptide **3** and the related guanosine-based tetrad at the basis of the secondary structure proposed by Boback et al. [90].

In addition, guanosine-containing nucleopeptides, such as the nucleo-tetrapeptide **3** (Figure 4), were reported to self-assemble into nanosheets and nanofibers [90]. Spectroscopic and microscopic analyses of the structures revealed that the peptide components of **3** drove the assembly into β-sheet structures, while hydrogen-bonded guanosines formed additional secondary structures synergistically within the peptide framework. The distinct supramolecular morphologies observed for this G-rich nucleopeptide self-assembly were not driven by metal cation responsiveness, as typically found in other guanine-based materials, but instead by the C-terminal peptide functionalization. This work allowed expanding the structural diversity of self-assembling nucleopeptides, showing new supramolecular applications for the guanosine-containing nucleopeptides [90].

Peculiar G-quadruplex formation has also been reported starting from guanosine-based amphiphiles, decorated on the sugar with both myristoyl groups and different polar chains, including oligoethylene glycol, amino acids and disaccharides [91]. The amino acid-derivatized lipidic guanosine derivatives proved to be good low molecular weight organogelators in organic solvents, such as methanol and ethanol, with promising antiproliferative activity on MCF-7 breast cancer cells in the low micromolar range [91].

**Figure 5:**
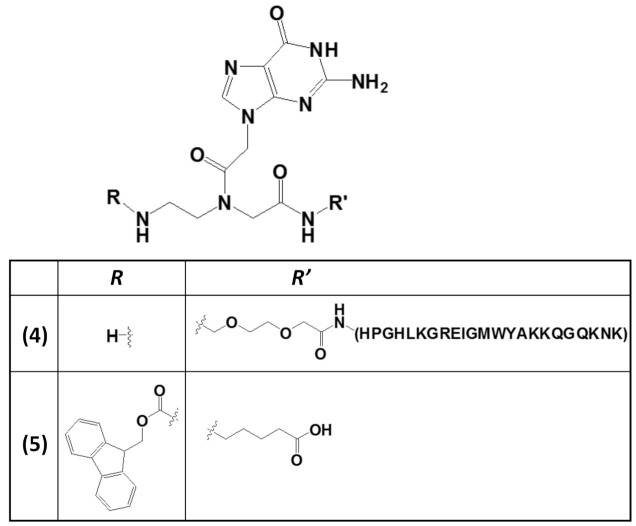
Chemical structures of two derivatives of a guanine PNA monomer involved in G4 formation: (i) the guanine PNA monomer covalently linked at the N-terminus of the RHAU23 peptide (**4**) [97] and (ii) the N-Fmoc-protected guanine PNA monomer amidated at the carboxylic function with a 5-aminopentanoic acid (**5**) [98].

The stabilization of G4 structures formed in G-rich nucleic acids by small-molecule ligands is considered an effective therapeutic approach for anticancer strategies [92,93,94,95]. This is particularly important in stabilizing specific G4-related structures, i.e., the G-vacancy-bearing G-quadruplexes (GVBQs) [96]. These are peculiar G4 structures containing a G-vacancy which can be stabilized by guanine-containing molecules, such as the physiological guanosine 5′-triphosphate GTP, by fill-in at the vacancy of a guanine nucleobase. GVBQs are involved in the regulation of enzymatic processes, including polymerase-catalyzed DNA synthesis. Moreover, since guanine derivatives are natural metabolites in cells, GVBQs can play an environment-responsive regulation role in cellular processes [96]. In this context, the RHAU23 peptide, functionalized at the N-terminus with a guanine moiety embedded into a guanine PNA monomer to form the nucleopeptide **4** (Figure 5), was selectively guided by the guanine unit toward a GVBQ, taking advantage of the filling-in of the G-vacancy in the nucleic acid target.

**Figure 6:**
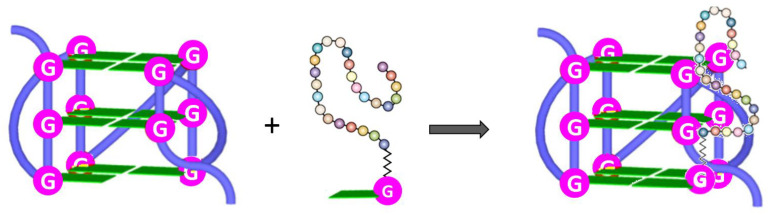
Schematic depiction of the interactions between G-nucleopeptides and GVBQs [97].

Furthermore, both the RHAU23 peptide and the guanine fill-in unit of **4** (Figure 6), interacting with the G-rich DNA, cooperatively improved the stabilization as well as the affinity toward the GVBQ. It is worth mentioning that targeting GVBQ DNA by this nucleopeptide system strongly suppressed in vitro DNA replication and RNA reverse transcription [97].

The wide applicability of DNA-based structures in nanotechnological strategies is partially impaired by their insufficient mechanical rigidity. To overcome this issue, the advantages of polyamide materials and the structural patterns inspired by nucleic acids were combined to generate a mechanically rigid fluorenylmethoxycarbonyl (Fmoc)-guanine PNA derivative (**5**, Figure 5) with specific morphology and photoluminescent characteristics [98]. The resulting G4-inspired mode of self-assembly led to a structure with each guanine head of one molecule hydrogen-bonded to the Fmoc carbonyl tail of another, generating a cation-free cyclic quartet arrangement. This structure was endowed with significant mechanical stability, as well as high mechanical stiffness, with Young’s modulus of 17.8 ± 2.5 GPa and an average stiffness of 69.6 ± 6.8 N m^−1^, which are values higher than those usually found for nucleic acid-derived structures. These properties were related to the head-to-tail packing and to additional π–π interactions mediated by the Fmoc moieties and the aliphatic chains [98].

## 4. Self-Assembling Nucleopeptides and PNAs in Biomedicine

**Figure 7:**
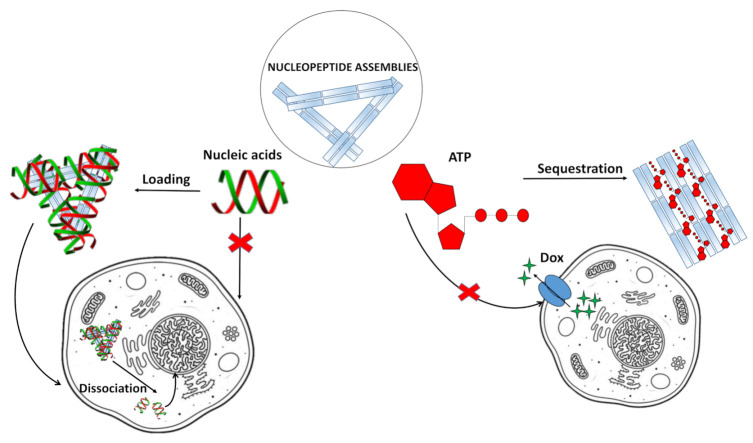
Examples of applications of nucleopeptide assemblies in biomedicine [43,110].

The self-assembly of bio-inspired nanomaterials and biological nanostructures confers new properties and functions to conjugated biomaterials, such as the ability to respond to external stimuli [99,100,101]. Nucleobase-containing peptides can be considered as aromatic peptides and, similar to these self-assembling structures [102,103,104,105,106], they can lead to interesting functional nanostructures as hereafter explained. Self-assembling nucleopeptides can form hydrogels based on supramolecular structures held by non-covalent molecular interactions occurring between the peptide segments, as well as π–π stacking and Watson–Crick interactions via complementary DNA bases. The ability of nucleopeptides and PNA to form highly ordered architectures has been recently exploited by the scientific community to develop controlled supramolecular tools such as nanotubes, nanovesicles, nanofibers, nanospheres, or micelles (e.g., spherical, cylindrical or worm-like), with applications in biomedicine, nanotechnology or materials science thanks to their biocompatibility and biodegradability (Figure 7) [107,108,109].

**Figure 8:**
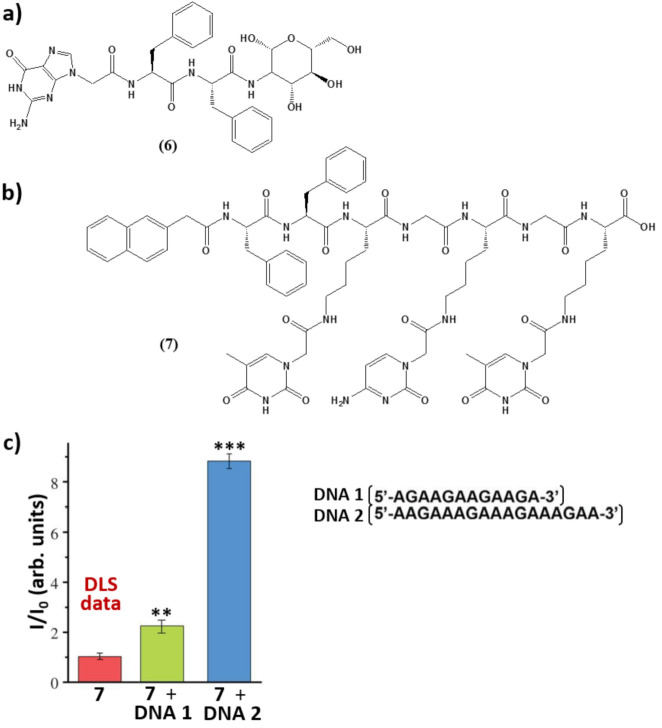
(**a**,**b**) Chemical structures of the self-assembling nucleopeptides described in [111] and [112], respectively. (**c**) DLS intensity of **7** at a concentration of 500 µM, of **7** with **DNA 1**, and of **7** with **DNA 2** (**7**/DNAs = 1:1) at an angle of 90°. Data are shown as mean ± s.d. ** *p* < 0.01, *** *p* < 0.001 by Student’s t-test. *n* = 3 [112].

For example, taking advantage of non-covalent interactions occurring between nucleic acids and nucleopeptides, it was possible to realize nucleopeptide-based supramolecular assemblies for gene release and therapy, able to selectively sequester ATP in cancer cells (Figure 7) increasing the efficacy of anticancer drugs [110], endowed with several unique benefits, i.e., i) reversible interactions between assemblies and nucleic acids, ii) minimal immunogenicity, and iii) biocompatibility. As shown by Li. et al. [111], the simple integration of nucleobase, amino acid, and glycoside moieties in one molecule via covalent bonds to form the nanostructured matrices of supramolecular hydrogels turned out to be an effective approach to imparting hydrogels with both supramolecular orders and multiple functions. These hydrogelators, based on single nucleobase-bearing oligopeptides conjugated to sugar moieties such as **6** (Figure 8a), showed morphologies characteristic of nanofibers, which did not significantly inhibit the growth of mammalian cells. Notably, the inclusion of the glycoside part increased the material protease resistance. Moreover, cell experiments indicated that these molecules interacted with nucleic acids, facilitating the oligonucleic acids’ entry into cytosol and the nuclei of cells [111]. In addition, a thymine-cytosine-thymine (TCT) nucleopeptide (**7**, Figure 8b) was able to self-assemble in water, forming nanofibers of around 6 nm in width and resulting in a hydrogel at a concentration of 1 wt%. Specifically, the nucleopeptide interacted with single-stranded DNAs containing complementary bases (AGA boxes), which in turn enhanced the self-assembly of the nucleopeptide, increasing the rigidity of the supramolecular hydrogel; these results emerged, among others, from CD and dynamic light scattering (DLS) data (Figure 8c) [112]. The same study also evidenced the ability of the nucleopeptide to interact with plasmid DNA and to deliver hairpin DNA into cells, as demonstrated by in vitro experiments. Overall, these results fully sustained the utility of nucleopeptides as soft biomaterials [112].

**Figure 9:**
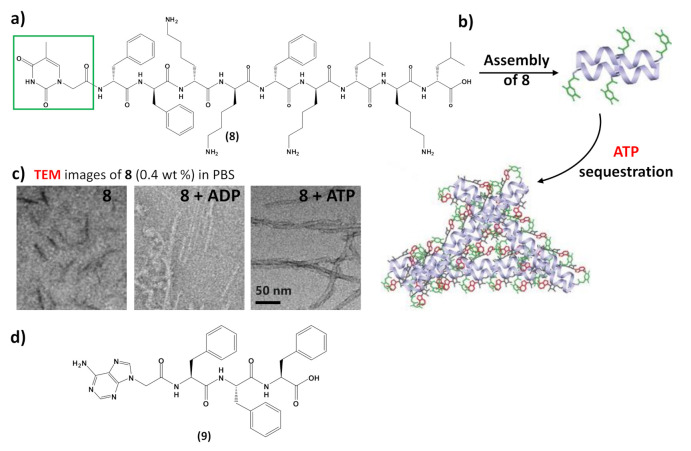
(**a**) Chemical structure of the self-assembling nucleopeptide **8** [43]; (**b**) schematic representation of ATP sequestration through nucleopeptide assembly, adapted with permission from [43]; (**c**) TEM images of **8** without or with 1 equivalent of ADP or ATP, adapted with permission from [43]; (**d**) chemical structure of the self-assembling nucleopeptide **9** [113].

In addition to interacting with nucleic acids, self-assembling nucleopeptides were used for sequestering cellular regulatory molecules and delivering anti-cancer drugs to tumors. In this regard, the work of Wang et al. [43] represents the first example of nucleopeptide assemblies that interacted with ATP, disrupting the intracellular ATP dynamics, a property exploited for controlling cell behavior. In detail, the study indicated that a D-nonapeptide (ffkkfklkl, f=D-phenylalanine, k=D-lysine, and l=D-leucine) conjugated to a thymine nucleobase at N-terminus through a carboxymethylene linker (**8**, Figure 9a) was able to selectively sequester ATP, discriminating ADP, in complex media like serum or cytosol. The nucleopeptide contained an ff box for increasing self-assembling ability, various lysines for interacting with the phosphate group of ATP, D-amino acids for proteolytic resistance, and the thymine to ensure specific Watson–Crick base pairing to the adenine of ATP. Nucleopeptide **8** formed a clear solution in PBS and serum, which also remained transparent in the presence of ADP, whereas it formed a precipitate after the addition of ATP (Figure 9b). As revealed by TEM, **8** formed short nanofibers with a length of 40 ± 5 nm and width of 4 ± 2 nm, which, in the presence of ATP, turned into uniform nanofibers of several hundred nanometers in length and 7 ± 2 nm in width (Figure 9c), which likely further aggregated to form the precipitate. ADP interacting with **8** only resulted in short nanofibers with diameters of 5 ± 2 nm (Figure 9c), which remained soluble. The nucleopeptide-based molecular system was proved to delay the efflux pumps in cancer cells by effectively sequestering ATP in cells, thereby increasing the efficacy of the DNA-intercalating chemotherapeutic, doxorubicin (Figure 7, right).

Moreover, nucleopeptide supramolecular assemblies can effectively deliver doxorubicin in a sustained manner, as proven by delivering the nucleopeptide-doxorubicin complex locally to a solid tumor [113]. In this approach, an adenine-bearing triphenyl alanine (**9**, Figure 9d) was used to form hydrogels able to load doxorubicin at high concentration, showing a continuous drug release under the in vitro degradation conditions exploited in the study [113]. In tumor-bearing mice, the doxorubicin-containing nucleopeptide hydrogels reduced tumor growth, resulting in higher apoptosis-mediated cell death in the tumor. Owing to the pharmacokinetic and biodistribution characteristics, the drug delivery by the nucleopeptide hydrogel improved and sustained delivery to the local tumor site [113].

Nucleopeptide constituents, i.e., nucleoamino acids, were shown to self-assemble in supramolecular structures, as seen in the case of spinacine and phenylalanine nucleoamino acids [7,114], in analogy to other small molecules [115,116]. Short nucleopeptides such as nucleobase-containing diserine, diphenylalanine, and dityrosine were also proven to form self-assemblies characterized by circular dichroism spectroscopy and light scattering [15,117]. Nucleopeptide-based colloidal materials were prepared using nucleobase morpholino β-amino acids [40]. These were used as intermediates, together with phenylalanine residues, to form diphenylalanine peptides functionalized with adenine and thymine bases. The obtained nucleopeptide was able to aggregate, and showed enhanced photoluminescent properties, including phosphorescence emission and deep blue fluorescence. These nucleopeptides were proposed as promising building blocks of advanced functional materials with applications in the optoelectronic field and in biotechnology [40].

It should be noted that the vast majority of the self-assembling nucleopeptides investigated to date as hydrogelators, leading to hydrogels and nanofibers with high biostability, contain short peptide sequences [118,119,120,121]. However, nucleobase-containing polypeptides have been also reported to form supramolecular assemblies that are potentially useful as novel biocompatible and biodegradable materials [122,123,124]. Recently, a nucleobase-modified polylysine was designed and synthesized by the polymerization of lysine and successive modification with thymines [125]. The self-assembly of the nucleobase-containing polypeptide chains was driven by melamine molecules containing three thymine-like faces. Notably, the morphology of the resulting assemblies (fibrous or spherical) was finely modulated on the basis of the degree of thymine substitution. An additional interesting application of polypeptides involves the formation of supramolecular complexes, containing diaminopyridine-functionalized polypeptides, hydrogen bound to thyminylpyrene moieties. Indeed, these complexes were proved to be efficient dispersants of carbon nanotubes (CNTs) thanks to stacking interactions between pyrene moieties and CNTs, and can be exploited in biomedical applications for their optoelectronic properties [126]. Finally, due to the interest in fluorescent-organic materials for optical applications in optoelectronic devices and as fluorescence sensors, polypeptides have been also studied for their photophysical properties. In particular, triphenyl pyridine- or triphenylamine-functionalized polytyrosine showed a similar aggregation-induced emission (AIE) behavior, which was explained as a consequence of the restriction of the intramolecular rotation mechanism for chromophoric compounds when attached to the polypeptides featured by a rigid-rod conformation [127,128].

## 5. Conclusions

Both single- and oligo-nucleobase-bearing peptides are interesting, conjugated compounds whose self-assembling behavior is significant in numerous research fields, ranging from prebiotic chemistry to biomedicine and nanomaterials science. Indeed, these bioinspired compounds are able to self-replicate, as required for a prebiotic genetic material, and, in several cases, form supramolecular systems and hydrogels with unique characteristics for potential therapeutic and nanotechnological applications. When functionalized with guanine nucleobases, nucleopeptide assemblies were often governed by the propensity of guanines to form G-quartets, accompanied by non-covalent interactions involving the peptide segments embedded into the nucleopeptide structures. Due to their nucleobase-decorated nature, the main nucleopeptide targets are clearly natural nucleic acids, as ascertained in the case of different DNA structures (single-stranded, hairpin and plasmid). Nonetheless, G4 DNA stabilization was also achieved in fill-in strategies using guanine-peptide conjugates directed toward GVBQ DNAs, thus forming supramolecular G4 DNA/nucleopeptide structures. Self-assembling nucleopeptides were also found to be able to deprive cancer cells of their “energy currency”, i.e., ATP, rendering them weaker and more vulnerable to anticancer drugs. Moreover, thanks to their nucleic acid-like nature, these molecular biosystems were able to bind DNA intercalators, delivering them to cancer cells at doses effective for significant anticancer effects. Finally, owing to their nanotechnological importance, novel biomaterials based on nucleopeptides were developed, taking advantage of the unique properties derived from their chimeric nature comprising both nucleobases and peptide moieties. These nucleopeptide-based biomaterials are generally able to self-assemble, to form superstructures driven mainly by hydrogen bonds and other non-covalent interactions. In consideration of the increasing interest in the above aspects, we envisage that the nucleopeptide assemblies formed by peptides carrying both single- and oligo-nucleobase may be utilized as components in the design of novel, valuable bioinspired materials that are useful in both biomedical and nanomaterials sciences.

## Data Availability

No data available.
